# Not a lymph node: ultrasonographic identification of a double pyramidal lobe of the thyroid gland

**DOI:** 10.1530/ETJ-25-0348

**Published:** 2025-12-08

**Authors:** Ilaria Giordani, Gerasimos P Sykiotis

**Affiliations:** Service of Endocrinology, Diabetology and Metabolism, Lausanne University Hospital and University of Lausanne, Lausanne, Switzerland

**Keywords:** thyroid, ultrasound

The pyramidal lobe is a morphological variation of thyroid anatomy, representing residual thyroid tissue that remains at the caudal end of the obliterated thyroglossal duct, the embryological structure connecting the foramen cecum of the tongue to the thyroid isthmus. Its prevalence is variable among studies depending on the employed detection methods, as high as 60% in autopsy and surgical series (1). The anatomic origin, location, size, and shape of the pyramidal lobe vary widely (2).

Double thyroid pyramidal lobe is a rare anatomical variant. The first report was published in 2009 in a patient operated for multinodular goiter (3), and the first ultrasonographic description was in 2014 in a series of 416 ultrasounds (2). In the few cases subsequently reported, it was usually found postoperatively in the pathology specimen (4, 5). Recently, Yan *et al.* have underlined the importance of movie clips during routine thyroid ultrasonography, whereby pyramidal lobe detection increases from 39.5 to 49.7% (6).

We report the case of a 45-year-old woman who consulted for a second opinion following a thyroid ultrasound for clinical impression of goiter; a 12 mm thyroid nodule and a solid isoechoic median structure in upper neck level VI were described, raising suspicion of a metastatic prelaryngeal lymph node.

We performed ultrasound with an 18 MHz linear transducer and found two spongiform (very low risk) nodules. We also identified two pyramidal lobes: one spanning from the central part of the thyroid isthmus to the hyoid bone and the other from the left part of the isthmus to the upper part of the left thyrohyoid membrane (Fig. 1; Supplementary video 1 (see section on Supplementary materials given at the end of the article)). The latter corresponded to the level VI structure that had been mistaken for a suspicious lymph node.

**Figure 1:**
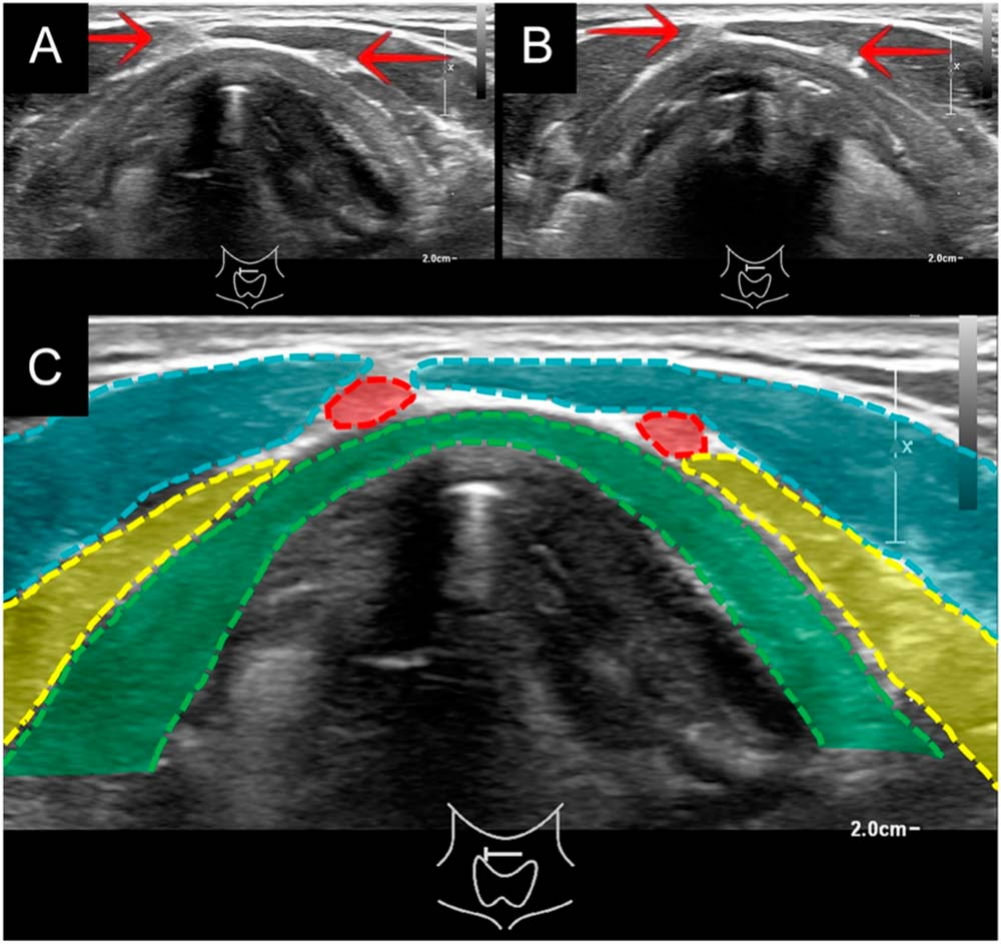
Transversal ultrasound images of the anterior neck. (A and B) The two pyramidal lobes (red arrows) are visible anterior to the thyroid cartilage at the level of the vocal cords (A) and slightly higher (B). (C) The anatomical structures shown in (A) are color-coded: two pyramidal lobes (red dashed lines), thyroid cartilage (green dashed lines), sternothyroid muscle (yellow dashed lines), and sternohyoid muscle (blue dashed lines).

Because the pyramidal lobe can be affected by all thyroid pathologies, its preoperative detection can have clinical consequences: it can be involved in any disease that affects the rest of the thyroid parenchyma, including nodules, cancer, and autoimmunity. This case highlights the importance of i) being aware of anatomical variations of thyroglossal remnants, ii) comprehensively scanning the upper level VI from the thyroid isthmus to the hyoid bone, and iii) correctly identifying the presence of pyramidal lobes and documenting them in ultrasound reports.

## Supplementary materials



## Declaration of interest

The authors declare that there is no conflict of interest that could be perceived as prejudicing the impartiality of the work reported.

## Funding

This work did not receive any specific grant from any funding agency in the public, commercial, or not-for-profit sector.

## Ethical approval

This work complies with the Swiss Federal Act on Research involving Human Beings. As confirmed by the research promoter’s office of our hospital, review by the Cantonal Research Ethics Committee for the Protection of Human Research Participants of the Canton of Vaud was not required. 

## Patient consent

Written informed consent was obtained from the patient for the publication of this case report.

## Author contribution statement

IG wrote the original draft. GPS conceived and supervised the study, validated the findings, and wrote and edited the manuscript.
